# Supplementary material for: Real-World Efficacy of Regdanvimab on Clinical Outcomes in Patients with Mild to Moderate COVID-19
Source: J Clin Med. 2022 Mar 4;11(5):1412. doi: 10.3390/jcm11051412 (PMC8911404; doi:10.3390/jcm11051412)
Supplement: Supplementary file 1 [file jcm-11-01412-s001.zip › jcm-1592572-supplementary.pdf]

Table S1. Sensitivity analysis of the impact of regdanvimab use on the initiation of remdesivir, dexamethasone, and oxygen supplementation in mild-to-moderate COVID-19 patients.

	Remdesivir			Dexamethasone			Oxygen supplement		
	**AOR**	**95% CI**	***P*-value**	**AOR**	**95% CI**	***P*-value**	**AOR**	**95% CI**	***P*-value**
**Regdanvimab**									
Users	0.095	0.024 - 0.373	0.001	0.089	0.016 – 0.477	0.005	0.078	0.015 – 0.420	0.003
Non-users	1			1			1		
**Age, years**	1.083	1.033 - 1.134	0.001	1.059	1.007 – 1.115	0.005	1.108	1.044 – 1.175	0.001
**Sex**									
Women	-			-			1		
Men	-	-	-	-	-	-	3.942	0.946 – 16.432	0.06
**BMI, kg/m^2**									
<25	1			1			1		
25 - <30	2.702	0.817 - 8.936	0.103	7.787	1.182 – 51.320	0.033	4.101	0.796 – 21.127	0.092
≥30	6.621	1.638 - 26.766	0.008	12.722	1.658 – 97.611	0.014	9.687	1.453 – 64.581	0.019
**Charlson comorbidity index**	-	-	-	-	-	-	-	-	-
**Vaccinated**									
Fully	-			-			-		
Not fully	-	-	-	-	-	-	-	-	-
**Initial body temperature**	4.145	1.941 - 8.854	<.001	2.559	1.017 – 6.443	0.046	2.711	1.107 – 6.637	0.029
**Pneumonia at admission**									
No	1			1			1		

Yes	10.287	2.850 – 37.133	<.001	19.391	1.878 – 200.252	0.013	26.696	2.618 – 272.199	0.006

Variable selection in the sensitivity analysis was performed under the backward elimination process using the likelihood ratio test. AOR = adjusted odds ratio; CI = confidence interval; BMI = body mass index.

Table S2. Safety profiles of laboratory results according to the usage of regdanvimab (CT-P59) using the linear mixed-effect model.

	**Users** a)			**Non-users** a)			
	**Baseline (HD 1)**	**HD 4**	**HD 7**	**Baseline (HD 1)**	**HD 4**	**HD 7**	P_{int} **b)**
CRP	2.0 (1.5 - 2.5)	1.6 (1.1 - 2.1)	0.6 (0.1 - 1.1)	2.0 (1.4 - 2.6)	1.7 (1.1 - 2.3)	1.1 (0.5 - 1.7)	0.617
WBC	5.2 (4.5 - 5.5)	5.1 (4.6 - 5.5)	6.5 (6.1 - 6.9)	5.0 (4.5 - 5.5)	5.1 (4.5 - 5.6)	6.2 (5.6 - 6.7)	0.529
Neutrophil	3.1 (2.6-3.4)	2.6 (2.3 - 3.0)	3.8 (3.4 - 4.1)	3.0 (2.6 - 3.4)	2.9 (2.5 - 3.3)	3.6 (3.1 - 4.0)	0.182
Lymphocyte	1.5 (1.4 - 1.7)	1.9 (1.8 - 2.1)	2.1 (1.9 - 2.2)	1.4 (1.2 - 1.6)	1.8 (1.6 - 2.0)	2.0 (1.8 - 2.2)	0.794
Hb	14.4 (14.2 - 14.7)	13.8 (13.5 - 14.0)	13.9 (13.6 - 14.1)	14.3 (14.0 - 14.6)	13.8 (13.5 - 14.2)	13.9 (13.6 - 14.2)	0.324
Platelet	202.6 (188.8 - 216.5)	211.7 (197.9 - 225.5	274.0 (260.2 - 287.9)	214.2 (197.2 - 231.2)	216.1 (199.1 - 233.1)	264.6 (247.6 - 281.6)	0.138
BUN	12.2 (11.5 - 12.9)	11.6 (10.9 - 12.3)	12.6 (11.9 - 13.3)	12.5 (11.6 - 13.3)	12.3 (11.4 - 13.1)	12.9 (12.0 - 13.7)	0.597
Creatinine	0.79 (0.76 - 0.81)	0.72 (0.70 - 0.75)	0.71 (0.69 - 0.74)	0.79 (0.74 - 0.80)	0.73 (0.70 - 0.76)	0.71 (0.68 - 0.74)	0.558
eGFR	103.6 (99.8 - 107.4)	115.5 (111.7 - 119.3)	115.3 (111.5 - 119.1)	106.5 (101.9 - 111.2)	113.8 (109.2 - 118.5)	116.5 (111.8 - 121.2)	0.143
Sodium	139.7 (139.3 - 140.0)	140.6 (140.2 - 141.0)	140.0 (139.6 - 140.4)	139.5 (139.0 - 140.0)	139.7 (139.2 - 140.2)	139.6 (139.1 - 140.1)	0.069
Potassium	4.1 (4.0 - 4.1)	4.2 (4.1 - 4.2)	4.3 (4.2 - 4.3)	4.1 (4.0 - 1.2)	4.1 (4.0 - 4.2)	4.2 (4.1 - 4.3)	0.11
Chloride	102.2 (101.7 - 102.7)	104.1 (103.6 - 104.6)	103.5 (103.0 - 104.0)	102.0 (101.4 - 102.5)	103.0 (102.4 - 103.6)	103.0 (102.4 - 103.6)	0.046
Calcium	8.4 (8.3 - 8.5)	8.3 (8.2 - 8.4)	8.5 (8.4 - 8.6)	8.3 (8.2 - 8.4)	8.4 (8.3 - 8.5)	8.5 (8.4 - 8.6)	0.074
Uric acid	5.0 (4.4 - 5.1)	4.7 (4.3 - 5.0)	4.7 (4.3 - 5.0)	5.0 (4.7 - 5.3)	4.6 (4.3 - 4.9)	4.9 (4.6 - 5.3)	0.128
Albumin	4.54 (4.48 - 4.61)	4.10 (4.03 - 4.16)	4.16 (4.10 - 4.23)	4.40 (4.32 - 4.48)	4.05 (3.97 - 4.13)	4.06 (3.98 - 4.14)	0.038
AST	33.5 (25.8 - 41.2)	32.6 (24.9 - 40.3)	33.6 (25.8 - 41.3)	42.8 (33.4 - 52.3)	39.0 (29.5 - 48.4)	37.1 (27.7 - 46.6)	0.686
ALT	40.8 (32.7 - 49.9)	41.2 (32.1 - 50.2)	48.6 (39.5 - 57.8)	43.8 (32.6 - 55.0)	41.3 (30.1 - 52.5)	44.0 (32.8 - 55.1)	0.517
ALP	218.3 (203.0 - 233.7)	201.8 (186.5 - 217.2)	215.3 (199.9 - 230.7)	204.4 (185.3 - 223.4)	195.8 (176.8 - 214.8)	203.4 (184.3 - 222.4)	0.111

aPTT	39.3 (38.2 - 40.4)	38.5 (37.9 - 39.6)	37.7 (35.6 - 37.8)	40.2 (38.8 - 41.6)	39.8 (38.4 - 41.2)	38.2 (36.9 - 39.6)	0.805
PT	12.6 (12.4 - 12.7)	12.7 (12.5 - 12.8)	13.0 (12.9 - 13.1)	12.9 (12.7 - 13.1)	13.1 (13.0 - 13.3)	13.4 (13.2 - 13.5)	0.346
LDH	223.8 (210.1 - 237.5)	207.3 (193.6 - 221.0)	203.1 (189.3 - 216.9)	227.8 (211.0 - 244.6)	221.5 (204.7 - 238.3)	221.9 (205.1 - 238.7)	0.422
GGT	48.3 (37.5 - 59.1)	50.7 (39.9 - 61.5)	55.2 (44.5 - 66.0)	53.8 (40.5 - 67.2)	55.9 (42.6 (69.3)	57.4 (44.0 - 70.7)	0.562
Ferritin	331.5 (236.7 - 426.3)	439.7 (346.1 - 533.3)	425.6 (329.9 - 521.3)	385.4 (266.0 - 504.8)	458.3 (339.9 - 576.7)	485.9 (366.7 - 605.0)	0.668

a) Estimates from the linear mixed-effect model were calculated after adjustment for covariates.

b) Based on repeated measures, the interactive effect of regdanvimab on the laboratory test results was calculated using the linear mixed-effect model.

Units for laboratory results: CRP (mg/dL), WBC (/μL), neutrophil (/μL), lymphocyte (/μL), Hb (g/dL), platelet (10^3/μL), BUN (mg/dL), creatinine (mg/dL), eGFR (ml/min/1.73m^2), sodium (mmol/L), potassium (mmol/L), chloride (mmol/L), calcium (mg/dL), uric acid (mg/dL), albumin (g/dL), AST (IU/L), ALT (IU/L), ALP (IU/L), aPTT (second), PT (second), LDH (IU/L), GGT (IU/L), and ferritin (ng/mL).

CRP = C-reactive protein WBC = white blood cell, Hb = hemoglobin, BUN = blood urea nitrogen, eGFR = estimated glomerular filtration rate, AST = aspartate transaminase, ALT = alanine transferase, ALP = alkaline phosphatase, aPTT = activated partial thromboplastin time, PT = prothrombin time, LDH = lactate dehydrogenase, GGT = gamma-glutamyl transferase, HD = hospitalization day, P_{int} = P-value for interaction.

Table S3. Changes in symptom severity score (SSS) according to the usage of regdanvimab (CT-P59) using the linear mixed-effect model.

	**Users** a)			**Non-users** a)			
	**Baseline (HD 1)**	**HD 4**	**HD 7**	**Baseline (HD 1)**	**HD 4**	**HD 7**	P_{int} b)
**Sum of SSS**	26.4 (24.9 - 27.7)	22.2 (21.0 - 23.3)	18.4 (17.3 - 19.6)	26.3 (24.9 - 27.7)	21.6 (20.2 - 22.9)	18.5 (17.1 - 19.9)	0.672
**Respiratory**	8.09 (7.67 - 8.5)	7.04 (6.27 - 7.46)	5.71 (5.29 - 6.12)	8.46 (7.95 - 8.97)	6.87 (6.37 - 7.46)	5.75 (5.24 - 6.26)	0.233
Runny nose	1.98 (1.82 - 2.13)	1.79 (1.63 - 1.94)	1.39 (1.24 - 1.55)	2.24 (2.05 - 2.43)	1.78 (1.59 - 1.97)	1.51 (1.32 - 1.7)	0.093
Sore throat	2.09 (1.93 - 2.24)	1.66 (1.5 - 1.82)	1.33 (1.17 - 1.49)	2.06 (1.87 - 2.25)	1.61 (1.42 - 1.81)	1.22 (1.02 - 1.41)	0.817
Shortness of breath	1.59 (1.45 - 1.73)	1.4 (1.26 - 1.54)	1.18 (1.03 - 1.32)	1.72 (1.54 - 1.89)	1.34 (1.16 - 1.51)	1.24 (1.07 - 1.42)	0.186
Cough	2.43 (2.27 - 2.6)	2.2 (2.03 - 2.36)	1.8 (1.64 - 1.97)	2.45 (2.25 - 2.65)	2.15 (1.95 - 2.35)	1.78 (1.58 - 1.98)	0.882
**Non-respiratory**	18.3 (17.5 - 19.2)	15.2 (14.3 - 16)	12.8 (11.9 - 13.6)	17.9 (16.8 - 18.9)	14.7 (13.7 - 15.8)	12.7 (11.7 - 13.8)	0.808
Fatigue	2.35 (2.18 - 2.53)	1.89 (1.72 - 2.07)	1.45 (1.28 - 1.63)	2.4 (2.19 - 2.61)	1.89 (1.68 - 2.1)	1.49 (1.28 - 1.7)	0.919
Myalgia	2.31 (2.15 - 2.47)	1.58 (1.42 - 1.74)	1.17 (1.01 - 1.32)	2.04 (1.85 - 2.23)	1.42 (1.23 - 1.61)	1.1 (0.91 - 1.29)	0.402
Headache	2.13 (1.96 - 2.29)	1.53 (1.37 - 1.7)	1.21 (1.04 - 1.37)	2.13 (1.93 - 2.33)	1.53 (1.33 - 1.73)	1.16 (0.96 - 1.36)	0.947

Chills	1.78 (1.64 - 1.92)	1.42 (1.28 - 1.56)	1.07 (0.93 - 1.21)	1.76 (1.59 - 1.93)	1.3 (1.13 - 1.47)	1.11 (0.94 - 1.28)	0.476
Feverish	1.98 (1.83 - 2.12)	1.48 (1.34 - 1.63)	1.17 (1.03 - 1.31)	1.77 (1.6 - 1.95)	1.31 (1.14 - 1.49)	1.15 (0.98 - 1.33)	0.383
Nausea	1.58 (1.43 - 1.72)	1.32 (1.17 - 1.46)	1.18 (1.04 - 1.33)	1.63 (1.46 - 1.81)	1.33 (1.15 - 1.51)	1.22 (1.04 - 1.39)	0.946
Vomiting	1.11 (1.04 - 1.18)	1.06 (1.0 - 1.13)	1.05 (0.99 - 1.12)	1.14 (1.06 - 1.22)	1.07 (0.99 - 1.15)	1.06 (0.98 - 1.14)	0.94
Diarrhea	1.61 (1.48 - 1.74)	1.39 (1.25 - 1.52)	1.26 (1.13 - 1.4)	1.64 (1.48 - 1.8)	1.27 (1.11 - 1.43)	1.3 (1.14 - 1.47)	0.287
Loss of smell	1.77 (1.59 - 1.94)	1.79 (1.61 - 1.96)	1.67 (1.49 - 1.84)	1.78 (1.56 - 1.99)	1.94 (1.72 - 2.15)	1.68 (1.47 - 1.9)	0.345
Loss of taste	1.71 (1.56 - 1.86)	1.67 (1.52 - 1.82)	1.51 (1.36 - 1.66)	1.62 (1.44 - 1.81)	1.69 (1.5 - 1.87)	1.46 (1.28 - 1.65)	0.547

a) Estimates from the linear mixed-effect model were calculated after adjustment for covariates.

b) Based on repeated measures, the interactive effect of regdanvimab on SSS was calculated using the linear mixed-effect model.

HD = hospitalization day, P_{int} = P-value for interaction.

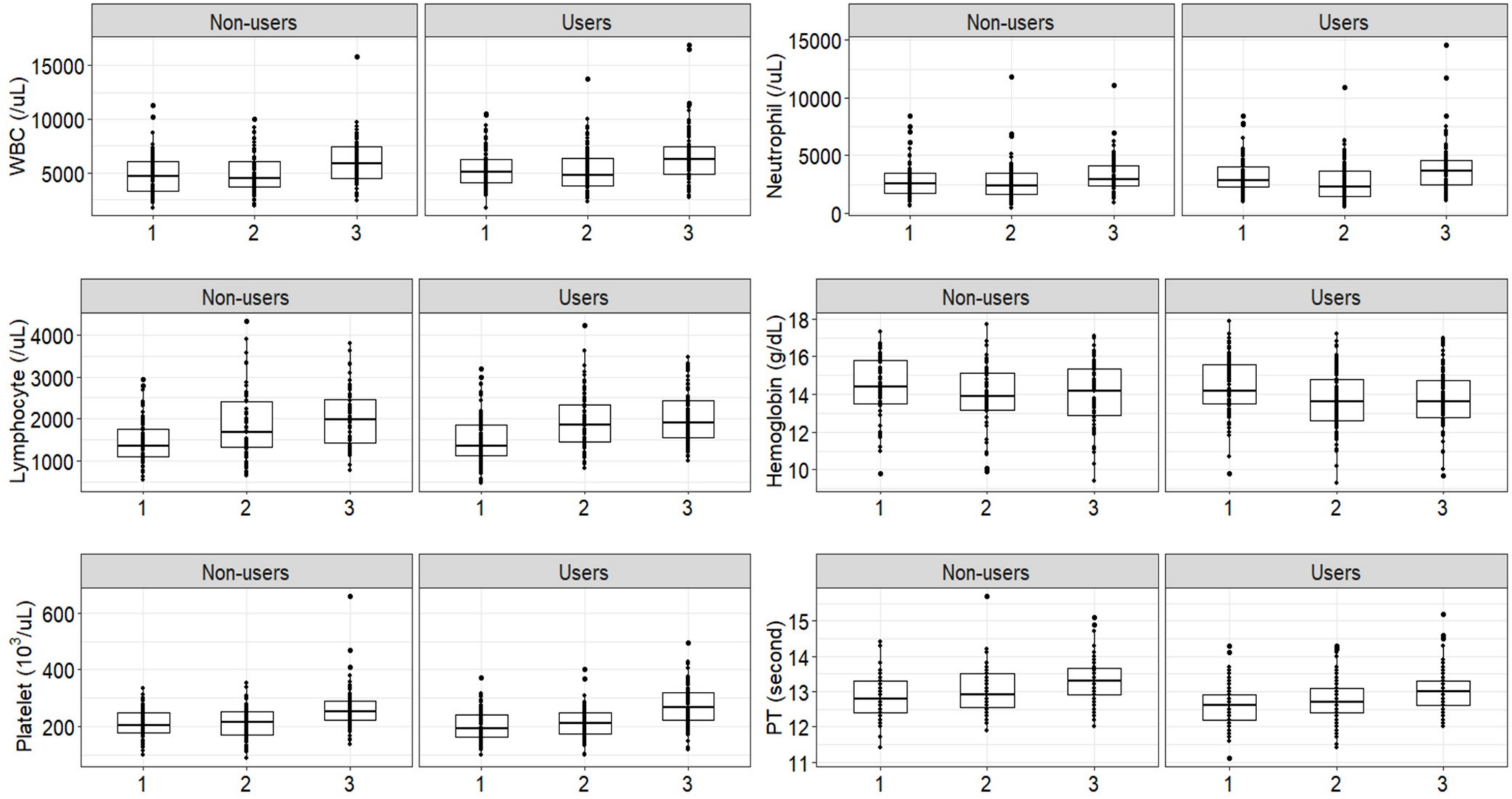

Figure S1. Changes in laboratory results of routine blood investigations and coagulation factors in patients receiving regdanvimab according to the time of the survey. HD = hospitalization day

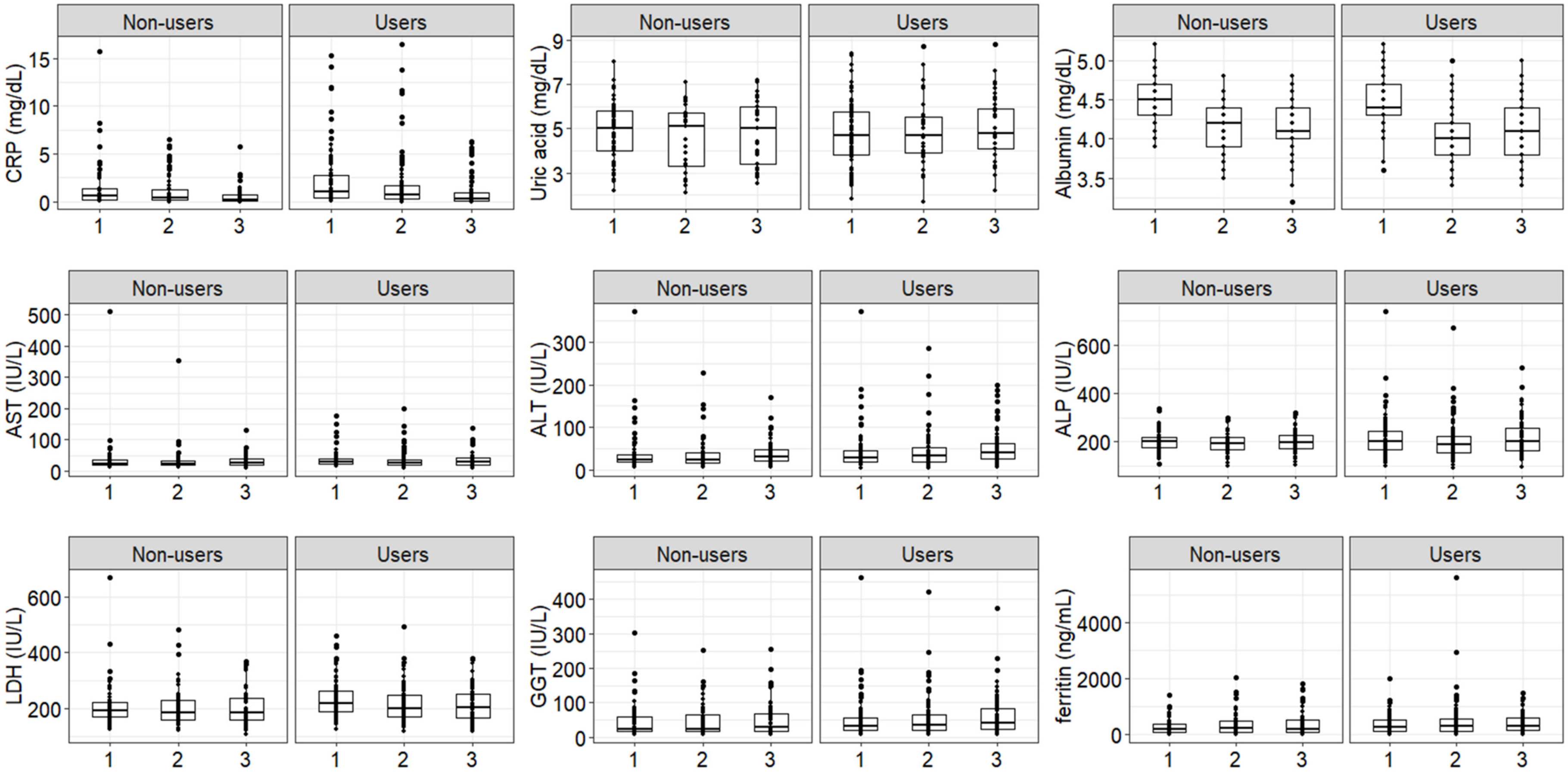

Figure S2. Changes in chemistry laboratory results in patients receiving regdanvimab according to the time of the survey. HD = hospitalization day

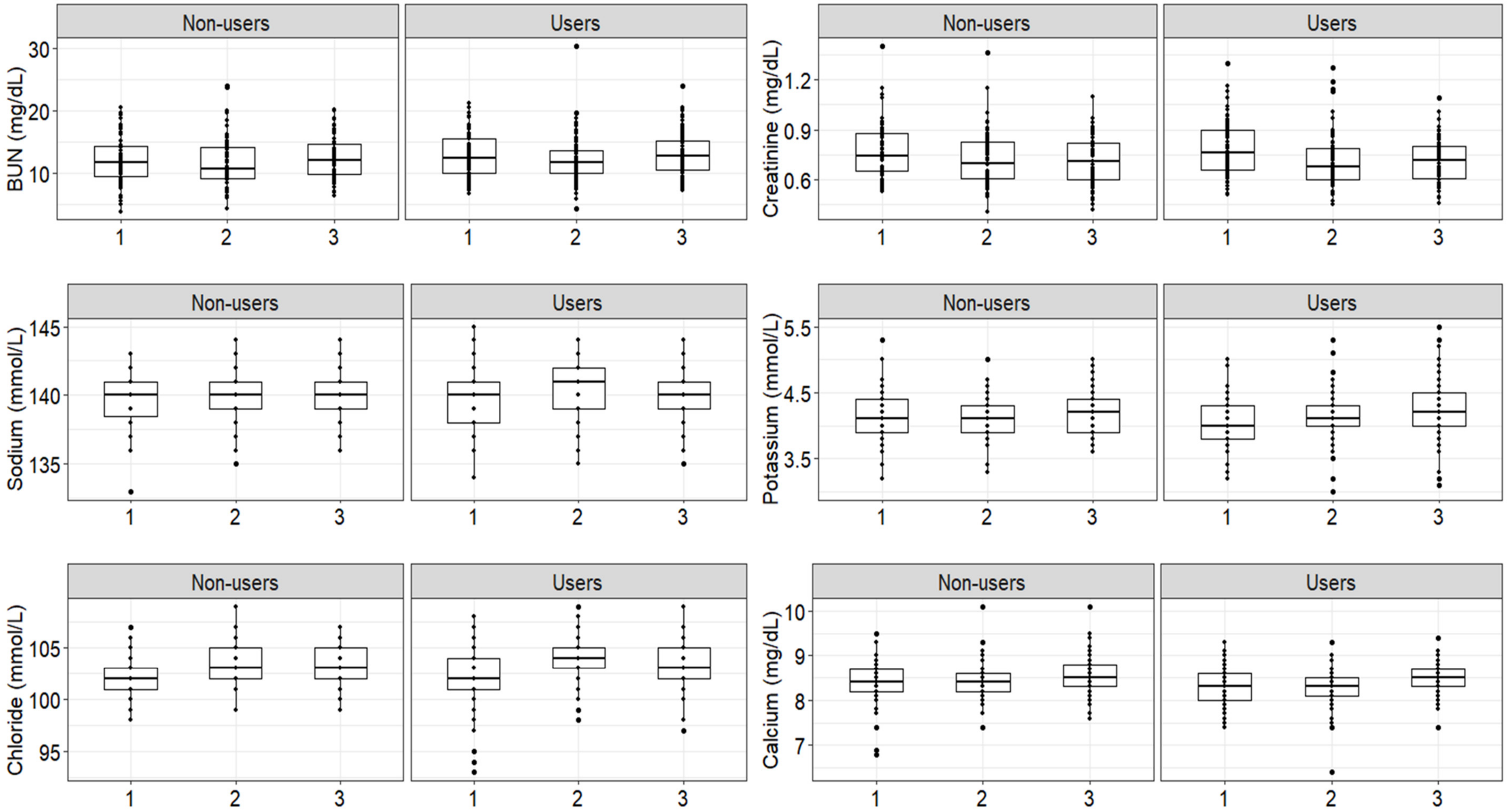

Figure S3. Changes in laboratory results of renal function in patients receiving regdanvimab according to the time of the survey: 1 (Baseline), 2 (1st follow up), and 3 (2nd follow up). HD = hospitalization day
